# Ensembl 2015

**DOI:** 10.1093/nar/gku1010

**Published:** 2014-10-28

**Authors:** Fiona Cunningham, M. Ridwan Amode, Daniel Barrell, Kathryn Beal, Konstantinos Billis, Simon Brent, Denise Carvalho-Silva, Peter Clapham, Guy Coates, Stephen Fitzgerald, Laurent Gil, Carlos García Girón, Leo Gordon, Thibaut Hourlier, Sarah E. Hunt, Sophie H. Janacek, Nathan Johnson, Thomas Juettemann, Andreas K. Kähäri, Stephen Keenan, Fergal J. Martin, Thomas Maurel, William McLaren, Daniel N. Murphy, Rishi Nag, Bert Overduin, Anne Parker, Mateus Patricio, Emily Perry, Miguel Pignatelli, Harpreet Singh Riat, Daniel Sheppard, Kieron Taylor, Anja Thormann, Alessandro Vullo, Steven P. Wilder, Amonida Zadissa, Bronwen L. Aken, Ewan Birney, Jennifer Harrow, Rhoda Kinsella, Matthieu Muffato, Magali Ruffier, Stephen M.J. Searle, Giulietta Spudich, Stephen J. Trevanion, Andy Yates, Daniel R. Zerbino, Paul Flicek

**Affiliations:** 1 European Molecular Biology Laboratory, European Bioinformatics Institute, Wellcome Trust Genome Campus, Hinxton, Cambridge CB10 1SD, UK; 2 Wellcome Trust Sanger Institute, Wellcome Trust Genome Campus, Hinxton, Cambridge, CB10 1SA, UK

## Abstract

Ensembl (http://www.ensembl.org) is a genomic interpretation system providing the most up-to-date annotations, querying tools and access methods for chordates and key model organisms. This year we released updated annotation (gene models, comparative genomics, regulatory regions and variation) on the new human assembly, GRCh38, although we continue to support researchers using the GRCh37.p13 assembly through a dedicated site (http://grch37.ensembl.org). Our Regulatory Build has been revamped to identify regulatory regions of interest and to efficiently highlight their activity across disparate epigenetic data sets. A number of new interfaces allow users to perform large-scale comparisons of their data against our annotations. The REST server (http://rest.ensembl.org), which allows programs written in any language to query our databases, has moved to a full service alongside our upgraded website tools. Our online Variant Effect Predictor tool has been updated to process more variants and calculate summary statistics. Lastly, the WiggleTools package enables users to summarize large collections of data sets and view them as single tracks in Ensembl. The Ensembl code base itself is more accessible: it is now hosted on our GitHub organization page (https://github.com/Ensembl) under an Apache 2.0 open source license.

## INTRODUCTION

Ensembl processes large-scale genomic data for chordate and model organisms, summarizes these data and provides powerful ways of accessing the information. Our annotations describe gene and transcript locations, gene sequence evolution, genome evolution, sequence and structural variants and regulatory elements. As of October 2014 (Ensembl release 77), we include full support for 69 species on our main website, plus partial support for 10 additional species on our Ensembl Pre! site (http://pre.ensembl.org). All of our data are provided without restriction from our websites, public MySQL databases, the Ensembl Application Programming Interface (API) and FTP site.

Data synchronization within and between species is important for algorithmic queries to run smoothly, and we achieve this by simultaneously releasing all our data in discrete, numbered releases. We maintain data consistency and traceability by processing our data with standardized pipelines that keep track of the raw evidence used to create our interpretations.

A large focus for this year was to update the data in Ensembl to the new human assembly, GRCh38. Below we include details of the efforts on GRCh38, as well as improvements in other parts of the project including updates to the Ensembl Tools server, Regulatory Build and outreach programme.

## NEW HUMAN ASSEMBLY - GRCh38

The Genome Reference Consortium (GRC) (1) released a new human genome assembly, GRCh38 (GCA_000001405.15), in December 2013. This is improved compared to GRCh37.p13, with new centromere representation, better segmental duplication accuracy, fewer assembly gaps and thousands of rare bases corrected (http://genomeref.blogspot.co.uk/2013/12/announcing-grch38.html).

In Ensembl, genes and transcripts are annotated by aligning protein and mRNA sequences to the genomic sequence. An updated human genome assembly is an opportunity to re-generate the annotation using the most updated supporting evidence for annotation. The re-analysis of the entire human genome annotation catalyses changes to all links from the annotation to other species in Ensembl, and to external resources such as the Universal Protein Resource (UniProt) (2) and HUGO Gene Nomenclature Committee (HGNC) (3). We recognize the potential impact on the wider community from the change and work to coordinate data flow between resources for both maximal usability and minimal confusion.

**Figure 1.**
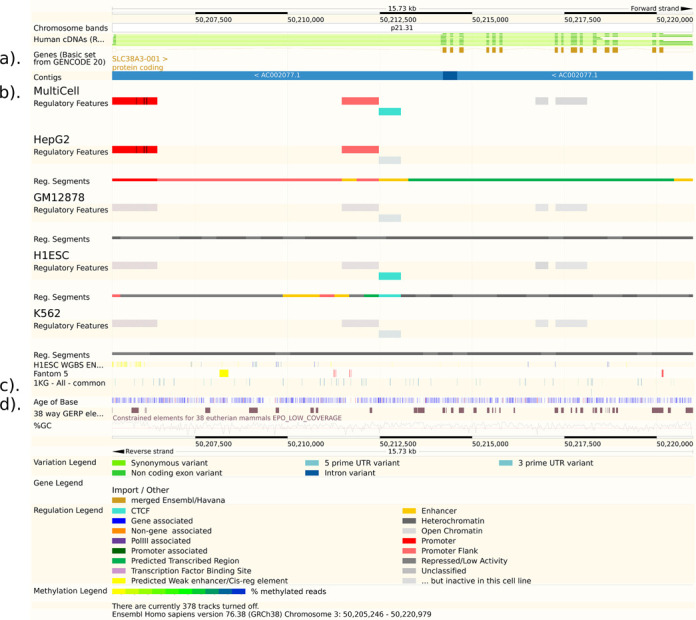
Ensembl ‘Region in Detail’ view showing the improved annotation of SLC38A3 in GRCh38, regulatory region information, default variation track and Age of Base track. (a) The GRCh38 assembly has resulted in an improved annotation of many genes compared to GRCh37.p13. Here we show the SLC38A3 gene as an example, where updates to the genome sequence now allow an open reading frame to be annotated. SLC38A3 is viewable in the GENCODE Basic track which shows only selected transcripts per gene. (b) The following regulatory tracks are shown from our new Ensembl Regulatory Build: MultiCell regulatory features (regions that are assigned a function that is independent of the cell type); cell type-specific regulatory data (for 4 selected cell types, out of 18 available - a regulatory feature is shaded in grey if it is inactive in the corresponding cell type, below each cell type is the cell type-specific segmentation track); H1ESC cell line Whole Genome Bisulphite Sequencing; Fantom5 (enhancers and promoters defined by the FANTOM5 project). (c) A track for variants genotyped by the 1000 Genomes project (phase 1) with frequency of at least 1% across any population is now on by default. The variants are colour coded by most severe consequence type (see Variation Legend in the lower part of the image). (d) Age of Base track: Each base pair that differs by a substitution in the human genome is classified as an event according to when it occurred: before the primate evolutionary branch (grey), in the primate-specific branch (blue), in the human-specific branch and is now fixed (red) or in the human-specific branch as a segregating variant (yellow).

The benefits of refreshing the gene set include removing gene models built from data that have since been discarded from the public databases, adding new isoforms that are supported by new data and annotation of new or improved genomic regions. In some cases genes that were annotated as non-coding in GRCh37.p13 are now coding in the new assembly (Figure 1a). A full Ensembl Gene set was produced on GRCh38, then merged with manual annotation from HAVANA to produce the GENCODE 20 gene set (4) and made available in Ensembl release 76 (August 2014). Regular updates of this manual annotation are planned in the coming year. For large consortia working on human data, we recommend using the GENCODE 21 gene set, made available in Ensembl release 77 (October 2014). In addition to updating the gene set, we recalculated pairwise whole-genome alignments from human to all other species in Ensembl and also our cross-species genome-wide multiple sequence alignments. The Regulation team regenerated regulatory annotation based on ENCODE (5) and Roadmap Epigenomics (6) data for the new assembly using a new Regulatory Build process, described below.

While re-analysis of data sets is generally preferable to get the most accurate results across the entire reference assembly (which includes new alternative loci and patched regions in GRCh38), it can be a time-consuming process. For some data sets a practical approach is to project the annotation for the areas of the genome where the genomic DNA remains unchanged. As a result, we have developed a method that produces a mapping between contig-based assemblies, such as GRCh37.p13 and GRCh38. The mapping is generated in three phases. Phase one identifies contigs that are shared, as well as being in the same order and orientation, between the two assemblies by comparing a contig's International Nucleotide Sequence Database Collaboration (INSDC) versioned accession or its sequence checksum. Phase two aligns any remaining unmapped gaps using LastZ (7). Phase three joins adjacent regions from phases one and two, and merges them to form the final assembly mapping that is stored in the Ensembl core database.

We use our assembly mapping data in the pipeline that assigns the stable identifiers for genes, transcripts, exons and proteins in GRCh37.p13 to their equivalent annotations in the new assembly. There are genes in GRCh37.p13 with two stable IDs: one for the gene model version annotated on the GRCh37 primary assembly and another for the different version of the gene on a fix patch. For these relatively rare cases we transferred the identifier from the GRCh37 primary assembly to the gene on GRCh38.

We also used the assembly mapping data to move variation annotation reliably from the older assembly to the newer one. For example, 98% of variants were projected successfully from GRCh37.p13 to GRCh38 and the remainder (in areas of significant assembly change) were re-aligned to the new assembly using Burrows-Wheeler Aligner (BWA). External user annotation can be projected via the Ensembl Perl API, the Ensembl REST API or as a tool on our website.

### Maintenance and updates for GRCh37.p13

As it will take time for various projects to update their analyses to the new assembly, we provide continued support for the latest GRCh37.p13 annotation via a new portal at http://grch37.ensembl.org. Programmatic API, MySQL and REST access are offered for data mining on the GRCh37.p13 data. We provide a full suite of tools, such as the Variant Effect Predictor (VEP) (8), BioMart (9) and Basic Local Alignment Search Tool (BLAST) (10) or BLAT (11) sequence search over the old GRCh37.p13 assembly. The website also provides links to equivalent regions in the new assembly from Gene pages, and Region in Detail via the left hand menu, making it easier to switch between the two resources.

The human gene set for GRCh37.p13 will be frozen as GENCODE 19 as no further manual annotation will be done on the previous assembly. We will update other data sets on the GRCh37 site, such as the 1000 Genomes Project (12) Phase 3 data on a 6–12 month update cycle for at least the next 2 years.

## UPDATED GENOMIC ANNOTATIONS

### Gene annotation

New fully supported species this year include: Olive baboon (*Papio anubis*), vervet/African green monkey (*Chlorocebus sabaeus*), Sheep (*Ovis aries*) (13), Amazon molly (*Poecilia formosa*), Mexican blind cave fish (*Astyanax mexicanus*) and Spotted gar (*Lepisosteus oculatus*). We update the annotation of supported species when significant new data, such as a new assembly, become available and this year we updated armadillo. All Ensembl Gene Builds over the last year have used Illumina RNA-seq data as supporting evidence, and we now have Binary Alignment/Map (BAM) files for these data available for over 20 species at ftp.ensembl.org. A new, matrix-based configuration tool has been introduced for RNA-seq data.

As mentioned above, automatic and manual annotation is merged to create the GENCODE gene sets for human and mouse. We also regularly update the zebrafish gene set to include manual annotation, and in Ensembl v77 (October 2014) created a merged manual and automatic rat gene set for the first time. To improve usability of the GENCODE gene set, we now identify a subset of representative transcripts for each gene as the GENCODE Basic set. GENCODE Basic prioritizes full-length protein coding transcripts over partial or non-protein coding transcripts within the same gene, and intends to highlight those transcripts that will be useful to the majority of users. These transcripts are identified in the transcript table on the Gene and Transcript summary view pages, and it is also possible to view the GENCODE Comprehensive and Basic gene sets as separate tracks in Region in Detail (Figure 1a). In addition to the GENCODE Basic flag, transcript support levels are also included for human and mouse and displayed in the transcript table. These values are computed by the University of California at Santa Cruz (UCSC) and indicate the level of support for each GENCODE transcript, thereby enabling users to sort and filter transcripts. We have also continued our long-running effort to produce consistent annotation among genome resources through the collaborative Consensus CDS (CCDS) project (14) with the aim of creating a core set of human and mouse protein coding regions that are consistently annotated and of high quality. We regularly compare the human and mouse GENCODE gene sets against National Center for Biotechnology Information (NCBI) RefSeq genes in order to produce the CCDS models.

### Regulatory region annotation

The Ensembl Regulation resources describe the dynamics of epigenetic marks across the genome and across cell types. The data cover 18 human and 5 mouse cell types, although these numbers will increase significantly in the next few months with the integration of more data from projects such as BLUEPRINT (15), ENCODE, Roadmap Epigenomics and HipSci (http://www.hipsci.org/). These features are annotated with transcription factor binding motifs from the 2014 release of the JASPAR database (16). Methylation is treated separately with 47 reduced representation bisulphate sequencing and whole genome bisulphate sequencing data sets on human, as well as links to external MeDIP data sets. We store external annotations, such as the VISTA enhancers, the FANTOM5 promoters (17) and enhancers (18) and the Diana TarBase miRNA target predictions (19).

To coincide with the annotation of the GRCh38 assembly, we inaugurated a new annotation algorithm (Ensembl Regulation Resources, in preparation) that computes regions of interest from genomic segmentations (20,21). In comparison to the previous Ensembl Regulatory Build (22), this new framework allows us to assign more precise functions to elements (e.g. enhancer, promoter) independently of cell type. A feature always retains its function, but a binary activity flag determines whether it is active or inactive in each cell type (see Figure 1b).

To allow for the projected increase in the number of described cell types, and to better highlight the new regulatory annotation, we redesigned the display of regulatory data. The aims of this redesign were to simplify the displays and also to increase discoverability, for example, by the use of icons to promote the different views available and new selectors for cell line and evidence type.

When selecting a regulatory feature, the user is first shown a page, which summarizes the genic context of that feature and its activity level across all cell types. The user can then request more detailed views of the data underlying the annotation, and access links to the original data source.

### Variation annotation

We have updated Ensembl to have the largest set of publicly available variation data with integrated phenotype data spanning 20 vertebrate species and the popular model organisms *Saccharomyces cerevisiae* and *Drosophila melanogaster*. This comprehensive knowledgebase is valuable as a platform for variation analysis. Our pipelines combine integrated quality control and annotation in order to connect data from different sources into one database per species (http://www.ensembl.org/info/genome/variation/sources_documentation.html). This results in a collection of over 323 million short sequence variants and indels with their associated allele frequencies and genotypes, 13 million structural variants, and phenotype and disease-associated annotation for 14 species. Both germline and somatic data are included but annotated as distinct data types. For any changes in gene or regulatory region annotation, we updated the variation consequence annotation so these data are consistent and we provided robust data synchronization for each Ensembl release. Ensembl is unique in providing programmatic access to genotypes and linkage disequilibrium information on a genome-wide scale (23) via the API. We also provide the Ensembl VEP for users to analyse their own data sets (see below).

We have developed infrastructure to display data from DECIPHER (24) and the Leiden Open Variation Database (LOVD) (25). These data are provided without bulk download facilities, as required by the access agreements for these projects. This year we added links across all species on the Ensembl website to indicate the presence of phenotype data for a corresponding gene orthologue. Furthermore, there are variation tracks on by default on the website for all common variants (i.e. with a minor allele frequency of at least 1%) from the 1000 Genomes Project (see Figure 1c), for all high quality structural variants from the 1000 Genomes Project, and for all known phenotype-associated variants.

To allow quality filtering within our data sets, we developed a pipeline to indicate the evidence supporting each variant by annotating if it has been observed multiple times, mentioned in a publication, or has been validated by allele frequency, or discovery in a large-scale project (e.g. the 1000 Genomes, Exome Sequencing Project (http://evs.gs.washington.edu/EVS/), HapMap Project (26)). We extended the variant citation information using data from the Genocoding Project (http://text.soe.ucsc.edu/) and from Europe PubMed Central (27) to provide further information, such as potential disease association, from the literature.

### Comparative annotation

Alongside our work on the new human assembly we have developed several new visualizations of comparative genomics data sets and these are available on the Ensembl website. A new resource called ‘Age of Base’ estimates the evolutionary timing of the most recent mutation within the human genome by using our 38-way mammalian whole genome alignment and is displayed as a track (see Figure 1d). Secondary structures of non-coding RNAs, based on information from RFAM if available (28) or RNAFold (29), can now be displayed. We overlay regions of highly conserved residues using sequence conservation statistics from the multiple alignments of our gene families. Our Enredo-Pecan-Ortheus (EPO) (30) whole genome multiple sequence alignments can now be visualized alongside the phylogenetic tree that describes the history of the aligned sequences.

We extended the classification of homologues in our system to cater for the inclusion of the wheat subgenomes into Ensembl Genomes Plants (31). Wheat has a hexaploid genome that is the product of the hybridization of three diploid species: we allow for the inclusion of each of these three subgenomes in the gene trees, and have introduced the term homoeologs to explain the relationship between them.

## ENSEMBL TOOLS AND INFRASTRUCTURE

Our new tools infrastructure was released this year. Ensembl Tools is based on eHive (32), our in-house compute farm management software, which vastly improves our ability to process larger data sets. Our VEP, BLAST or BLAT sequence search, and assembly converter tools were redeveloped to run on this new infrastructure, and all of these tools now also have refreshed web interfaces. Users can save job results if they are logged into the system. Usability has been a focus for improvement within the project and has contributed to enhancements in these new tools and a more streamlined sequence export from the browser.

To provide a faster sequence search, we migrated from WU-BLAST to NCBI BLAST+. We also replaced our assembly converter tool with one based on CrossMap (33) to enable conversion of a wider set of formats and support for both Ensembl and UCSC chromosome naming conventions.

### VEP

**Figure 2.**
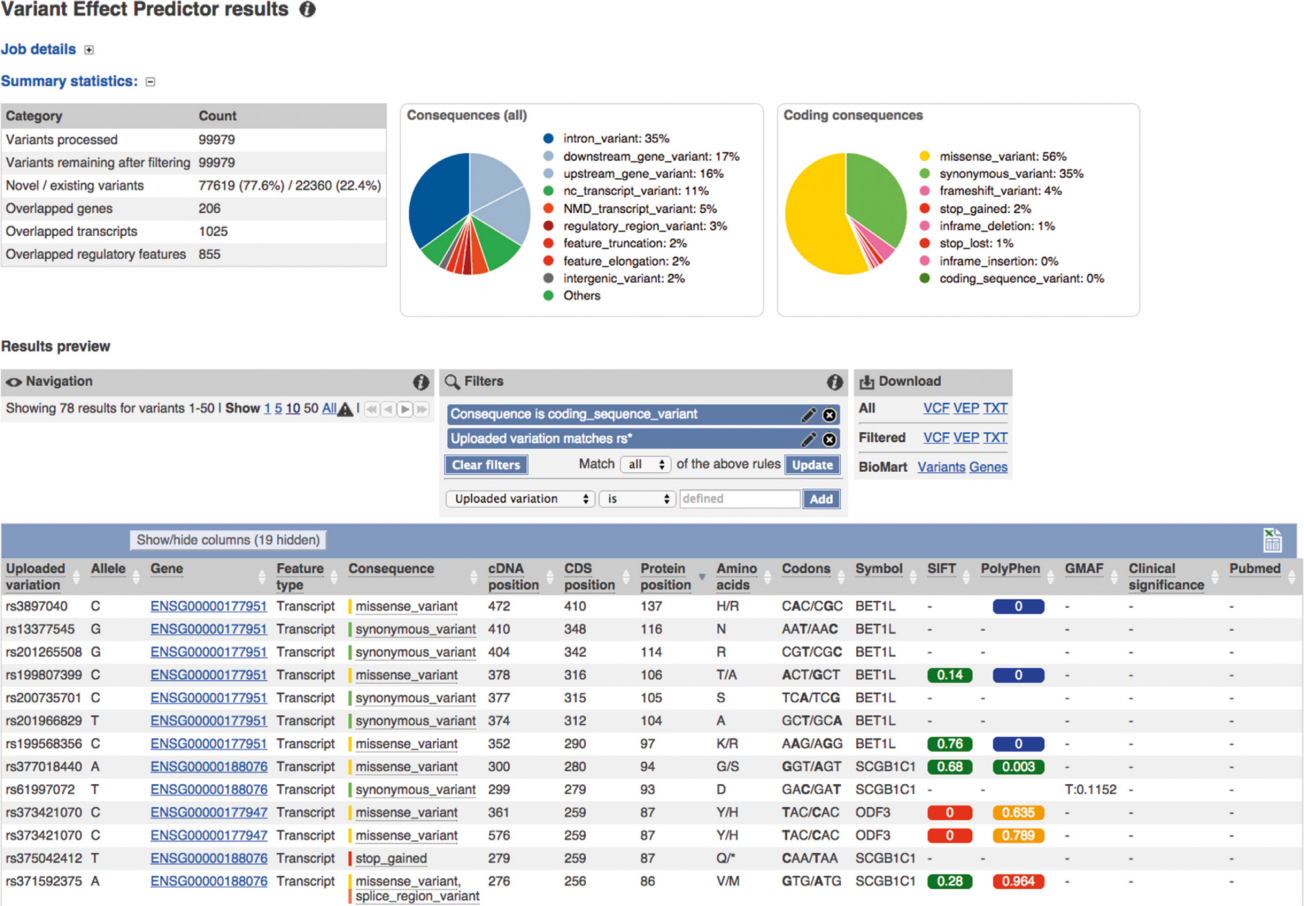
Improved VEP web tool and interface: The output is summarized using pie charts and statistics. Additionally, the results preview can be filtered on any output column. The output data can be filtered and downloaded for further analysis.

This year we released several updates to the Ensembl VEP, a powerful and flexible tool for users to annotate their own variation data. VEP can be applied to all Ensembl supported species and even species not supported by Ensembl: it only requires a genome FASTA file and transcript annotation in a GTF file. As noted above, the new Ensembl Tools-based infrastructure includes a VEP interface providing summary output including statistics, pie charts and a data table that are sortable and filterable (Figure 2). The VEP off-line script version has been extended to include additional data types (PubMed IDs, Exome Sequencing Project frequencies, UniProt IDs, HGNC IDs) with new plugins for popular data sets, such as FATHMM (34), dbNSFP (35) and CADD (36). VEP can now annotate on both RefSeq and Ensembl transcripts, as well as the GENCODE Basic transcript set and Locus Reference Genomic sequences (37,38) or a user's own custom gene set. With the improvements to the REST API (described below) and web tools infrastructure, an entire human variome (∼3 million variants) can be processed in a couple of hours without the need for large client computers.

### REST service

Ensembl's REST service (39) transitioned from beta to a fully supported service hosted at http://rest.ensembl.org. The new server brings an increase in processing capacity as well as a higher rate-limit, from 3 requests per second to 15 per second. It also provides HTTPS access for secure web applications. We continue to develop and expand our range of endpoints which now include EPO genomic alignments and gene gain/loss trees in JSON and PhyloXML formats, as well as query support for archived identifiers. To cater for an increasing number of users, we provide POST support for a number of endpoints, which allows a user to submit queries as one large batch rather than individual requests. For example, for the VEP, users can submit up to 1000 variants in one request (i.e. 15 000 variants per second) a 5000-fold increase over the VEP annotation rate on the beta server. The REST API's documentation has received a major overhaul including a comprehensive user guide hosted on our GitHub wiki (https://github.com/Ensembl/ensembl-rest/wiki).

### eHive

eHive, our jobs submission tool, has received three major updates. First, we have released guiHive, a graphical interface that allows the user to monitor running pipelines and perform common operations without using the MySQL command line. Secondly, version 2.0 no longer depends on the Ensembl core API thereby making eHive a standalone tool. Finally, we have developed a utility to report compute farm resource usage that can be used to tune pipelines, for example, by identifying if the requested memory or runtime resources are in line with those used.

### WiggleTools

We developed WiggleTools (40) to take in a collection of genome-wide data sets stored in BigWig, BigBed (41) or BAM files, and produce a summary track that can be viewed in Ensembl. WiggleTools offers a range of statistics, such as mean, median, minimum, maximum or variance and allows the users to compare groups of data sets. For example, the mean of a collection of case and control replicates can be compared in addition to more advanced statistics, such as Welch's T-test (for normally distributed variables) or Wilcoxon's rank sum test (for other variables). Additionally, a novel BigWig file merging tool, bigWigCat, which we contributed to the UCSC command-line genomics utilities library (http://hgdownload.soe.ucsc.edu/admin/exe/), allows WiggleTools to make the most of a cluster of computers. For example, to compute the sum of 126 BigWig files (a total of 121 GB) takes less than 17 min in total, on 116 CPUs, and fits on less than 5.5 GB of RAM.

### BioMart

The Ensembl BioMart continues to be updated with each Ensembl release and remains our primary data-mining tool. Users can now query for phenotypes linked to genes and can output the transcript annotation source in their queries. Furthermore, our regulation marts have been rebuilt to provide access to the new regulatory annotation. BioMart continues to be accessible from our website, while programmatic access is available from BioMart's Perl, REST APIs and the popular BioConductor biomaRt package (42).

### Git

2014 saw Ensembl move from using CVS (Concurrent Versions System) to using Git, a modern decentralized version control system. Motivated by the collaborative nature of Git and enhancements over CVS, we migrated all repositories including their 14-year histories. To help users with this transition we continued to backport major releases and changes to CVS up to release 77. All Ensembl code is now hosted on GitHub. Git enables our developers to have more stable and efficient development practices for creating new features outside our release branches. It also allows our collaborators to contribute corrections and improvements to the code base without undue obstacles.

We use Travis-CI (https://travis-ci.org) to continuously integrate commits to a number of Ensembl repositories including those changes submitted by external contributors. Alongside this migration we developed two tools, hosted in our ensembl-git-tools project, to help internal and external users work with Git. The first, git-ensembl, allows a user to issue commands that operate over multiple repositories, e.g. switching branches in all Ensembl API checkouts. The second, git-mgw, provides a lightweight workflow for shared Git repositories that keeps development away from the master branch. Integration occurs just before sharing, encouraging a linear commit history. git-mgw can be used by any Git repository and provides an alternative to workflows, such as gitflow.

## OUTREACH AND TRAINING

Each year, Ensembl offers around 100 worldwide training events and supports more than 1500 user queries on our Helpdesk (helpdesk@ensembl.org) and Developer's lists (dev@ensembl.org). We offer online learning via the Ensembl Helpdesk Channel on YouTube, www.youtube.com/user/EnsemblHelpdesk (YouKu http://i.youku.com/u/UMzM1NjkzMTI0 for users in China) where we host over 20 videos, and through courses on the popular EMBL-EBI Train Online platform (currently over 1 million page views, 150 000 visitors), http://www.ebi.ac.uk/training/online.

Webinars have been used this year in both a training and dissemination capacity. We held eleven webinars: five as part of bioinformatics courses in Korea, Costa Rica, Puerto Rico and Pakistan, five linked to each Ensembl release, and a special topic webinar on the new human assembly, GRCh38, in conjunction with the GRC.

The user community can follow our news and upcoming developments via Twitter (@ensembl), our blog (http://www.ensembl.info) and Facebook (http://www.facebook.org/Ensembl.org). For example, we published 13 blog posts describing the progress and details of the new human assembly annotation (http://www.ensembl.info/blog/category/genebuild/grch38/). Our Twitter hashtags including #CitedEnsembl, #EnsemblVEP and #Ensembl76 make it simpler to track workflows using Ensembl and share developments by topic of interest.

On the Ensembl website there are new, context-specific Q&As and a new tutorials page that supports both new and existing users who want to deepen their understanding of Ensembl. These materials are also available for use by those running their own bioinformatics courses: http://www.ensembl.org/info/website/tutorials/index.html.
